# Mapping reservoir water quality from Sentinel-2 satellite data based on a new approach of weighted averaging: Application of Bayesian maximum entropy

**DOI:** 10.1038/s41598-024-66699-2

**Published:** 2024-07-16

**Authors:** Mohammad Reza Nikoo, Mohammad G. Zamani, Mahshid Mohammad Zadeh, Ghazi Al-Rawas, Malik Al-Wardy, Amir H. Gandomi

**Affiliations:** 1 Department of Civil and Architectural Engineering, Sultan Qaboos University, Muscat, Oman; 2 Department of Civil and Environmental Engineering, Colorado State University, Fort Collins, USA; 3 Department of Soils, Water, and Agricultural Engineering, Sultan Qaboos University, Muscat, Oman; 4 Faculty of Engineering and IT, University of Technology Sydney, Ultimo, NSW 2007 Australia; 5 University Research and Innovation Center (EKIK), Óbuda University, 1034 Budapest, Hungary

**Keywords:** Water quality assessment, Remote sensing, Sentinel-2, Bayesian maximum entropy-based fusion, Machine learning, Environmental monitoring, Computational science, Civil engineering

## Abstract

In regions like Oman, which are characterized by aridity, enhancing the water quality discharged from reservoirs poses considerable challenges. This predicament is notably pronounced at Wadi Dayqah Dam (WDD), where meeting the demand for ample, superior water downstream proves to be a formidable task. Thus, accurately estimating and mapping water quality indicators (WQIs) is paramount for sustainable planning of inland in the study area. Since traditional procedures to collect water quality data are time-consuming, labor-intensive, and costly, water resources management has shifted from gathering field measurement data to utilizing remote sensing (RS) data. WDD has been threatened by various driving forces in recent years, such as contamination from different sources, sedimentation, nutrient runoff, salinity intrusion, temperature fluctuations, and microbial contamination. Therefore, this study aimed to retrieve and map WQIs, namely dissolved oxygen (DO) and chlorophyll-a (Chl-a) of the Wadi Dayqah Dam (WDD) reservoir from Sentinel-2 (S2) satellite data using a new procedure of weighted averaging, namely Bayesian Maximum Entropy-based Fusion (BMEF). To do so, the outputs of four Machine Learning (ML) algorithms, namely Multilayer Regression (MLR), Random Forest Regression (RFR), Support Vector Regression (SVRs), and XGBoost, were combined using this approach together, considering uncertainty. Water samples from 254 systematic plots were obtained for temperature (T), electrical conductivity (EC), chlorophyll-a (Chl-a), pH, oxidation–reduction potential (ORP), and dissolved oxygen (DO) in WDD. The findings indicated that, throughout both the training and testing phases, the BMEF model outperformed individual machine learning models. Considering Chl-a, as WQI, and R-squared, as evaluation indices, BMEF outperformed MLR, SVR, RFR, and XGBoost by 6%, 9%, 2%, and 7%, respectively. Furthermore, the results were significantly enhanced when the best combination of various spectral bands was considered to estimate specific WQIs instead of using all S2 bands as input variables of the ML algorithms.

## Introduction

Urban areas threaten the purity of lakes and reservoirs because of the unauthorized release of industrial water and household sewage, which often contaminates these bodies of water^1,2^. The deterioration of water quality can have several detrimental effects, including increased human exposure to disease and harmful chemicals^3,4^, a decrease in ecosystem productivity and biodiversity^5^, and harm to water-related industries, such as aquaculture and agriculture^6^. Water quality monitoring has leaned on two approaches: producing water samples for analysis in a controlled setting or employing automated in situ measurement. However, such procedures can be labor-intensive or expensive^7^. Additionally, the majority of water sample analyses require regents for examination and managing the disposal of generated waste during the testing phase can be prohibitively expensive. While such methods may be highly accurate, individual samples only provide information on the quality of water at the very location in which water quality is measured, and they have limited ability to fully characterize the overall information of WQIs through the whole water bodies^8^. As a result, over time, there has been transition from conventional in situ measurement techniques towards the utilization of RS methods^9^. RS technology utilizes sensors that are either airborne or located in space to measure the radiation reflected across various wavelengths from the surface of a body of water, which can provide valuable information about its quality. The reflections can be employed either directly or indirectly to determine various WQIs^10,11^. To monitor and assess WQIs, it is crucial to consider several essential factors, including the spectral attributes of both the water and its contaminants. These characteristics are determined by the chemical, biological, and hydrological properties of water^12^.

The launch of the Multi-spectral Imager’s (MSI) onboard S2 in 2015 significantly enhanced the potential for RS of reservoirs. With spatial resolutions of 10, 20, and 60 m, the imagery allows for studying even small reservoirs. Moreover, data is required across 13 spectral bands, and the sensor’s radiometric resolution is 12-bit. The second satellite launched into orbit at the beginning of 2017, after which the revisit time of S2 was 5 days^13^. Although RS imagery can provide valuable information, meaning and analyzing large-scale datasets using traditional techniques can be challenging. Therefore, there has been a recent shift toward utilizing cutting-edge techniques, including incorporating ML models into geo-referenced databases, to improve the analysis and management of these data.

The emergence of ML algorithms, when combined with advanced data processing technologies and powerful computing capabilities, has opened up novel opportunities to decode, quantify, and grasp processes propelled by significant data volumes^14^. Combining ML with satellite RS data offers a powerful approach for the routine assessment of variance in WQIs across space and time. Such a procedure provides a viable method to incorporate water quality data obtained from conventional in situ measurements^15^. Furthermore, ML algorithms facilitate faster estimation of WQIs, enabling real-time measurements. Collaborating with RS imagery, these algorithms decrease the need for human involvement in analyzing vast amounts of data, delivering exceptional precision at a low cost^16^.

Recently, a large number of researchers have considered the application of ML algorithms to analyze RS satellite data. As a result, several prediction models, considering the combination of RS and ML algorithms, have been effectively used in recent decades to estimate and forecast water quality characteristics within bodies. These algorithms include artificial neural networks^17–21^, SVR^22–26^, random forest^27–35^, decision tree^36–41^, logistic regression, Naïve Bayes^42,43^, KNN^44–47^, and boosting algorithms^48,49^.

Guo et al. conducted a study wherein they assessed 255 different compositions of bands in S2 imagery to determine the optimal ones for retrieving different WQIs^50^. In Guo’s study, three ML algorithms, including RF, SVR, and NNs, were examined side by side to identify the best combination of ML and S2 satellite data. In another study, Adusei et al., focused on retrieving and mapping WQIs for the Owabi Dam reservoir by leveraging S2 and Landsat 8 satellite data. To accomplish the goal, the researchers employed three ML models, namely RF, SVM, and MLR^51^. The obtained results indicated that the S2 and RF models were recommended for monitoring the surface water quality of the study area. Tian et al. utilized S2 images to evaluate the performance of eXtreme Gradient Boosting, SVR, RF, and ANN in estimating different WQIs for inland reservoirs. The findings indicated that XGBoost performed better than the remaining three algorithms in terms of evaluation indices^52^. Tian et al. employed a combination of RS and ML algorithms to recover Chl-a, DO, and ammonia nitrogen levels in inland reservoirs. Their findings revealed that XGBoost demonstrated superior performance compared to alternative algorithms^53^. Between 2016 and 2018, more than 200 records of water quality data, encompassing blue-green algae phycocyanin (BGA-PC), Chl-a, DO, specific conductivity, fluorescent dissolved organic matter (fDOM), turbidity, and pollution sediments. Their findings underscored the significant potential of both proximal and satellite-based sensors in accurately estimating optically active parameters. However, while remote sensing may indirectly estimate non-optically active parameters, it continues to pose challenges^54^. Leggesse et al., formulated six ML models that incorporated Landsat 8 imagery to assess their precision in forecasting three optically active WQIs monitored on a monthly basis from August 2016 to April 2022. Their research demonstrated the feasibility of monitoring water quality in extensive freshwater bodies with sparse observed data through the integration of RS and ML algorithms, potentially augmenting decision-making processes^55^.

In addition to ML techniques, the employment of ensemble regression or decision-level fusion has garnered significant interest among the RS community^56,57^. In a regression problem, decision-level fusion refers to the amalgamation of multiple models and their outputs to generate a single model. This approach is widely preferred because it exploits individual strengths and reduces the biases inherent in employing a single modeling technique^58^. According to Liu et al.^59^ decision-level fusion can offer more consistent predictions in regression problems, and its adoption may also enhance the transferability of models while capturing diverse correlations that may not be evident in models dependent on a solitary regression technique. It has also been discovered that the amalgamation of various ML algorithms can aid in crafting accurate models for estimating and predicting WQIs^60^.

The research indicates that the BMEF procedure, which is one of the procedures to fuse various ML algorithms, plays a vital role in combining individual ML algorithms. However, RS has not yet been used coupled with ML and the BMEF concept to improve the outcome generated by predictive models for WQIs. Therefore, a BME-based fusion model is proposed to estimate the outcomes generated by individual ML algorithms combined with RS satellite data. To put it differently, the main aim of the BMEF procedure is to combine the output of many estimation or predicting models in order to take advantage of the strengths and capabilities of each model^61^. As a result, the more similar a prognosis is to the relevant observation, the greater the models’ weights in the process of fusion of individual models^62^.

This research introduced an innovative framework for contrasting singular ML algorithms, such as MLR, RFR, SVR, and XGBoost, and their fusion based on BMEF. Although several ML and DL estimation procedures have been utilized in the previous research for estimating and predicting WQIs, as far as the authors know, such researches lack a comparison of ML models along with their fusion based on BMEF regarding RS satellite data. Therefore, this research addressed the gap in knowledge by evaluating the precision of four distinct and widely recognized ML algorithms and their fusion. The intended purpose of the suggested framework was to:

1. Creating dataset based on field measurement, using a CTD sensor, and satellite-based data using S2
2. Developing four individual ML algorithms to estimate WQIs
3. Examining the effectiveness of ML models based on various evaluation indices to compare the outcomes of ML models together
4. Developing a new hybrid model based on the weighted averaging method, considering uncertainty
5. Comparing the results generated by individual ML algorithms and those of the BMEF model

The benefits of BMEF models for estimating WQIs using RS data include a wide range of stakeholders, such as water utilities, environmental agencies, policymakers, and the general public. For instance, water utilities can use these models to optimize their treatment processes, reduce costs, and ensure compliance with regulations.

## Materials and methods

**Figure 1:**
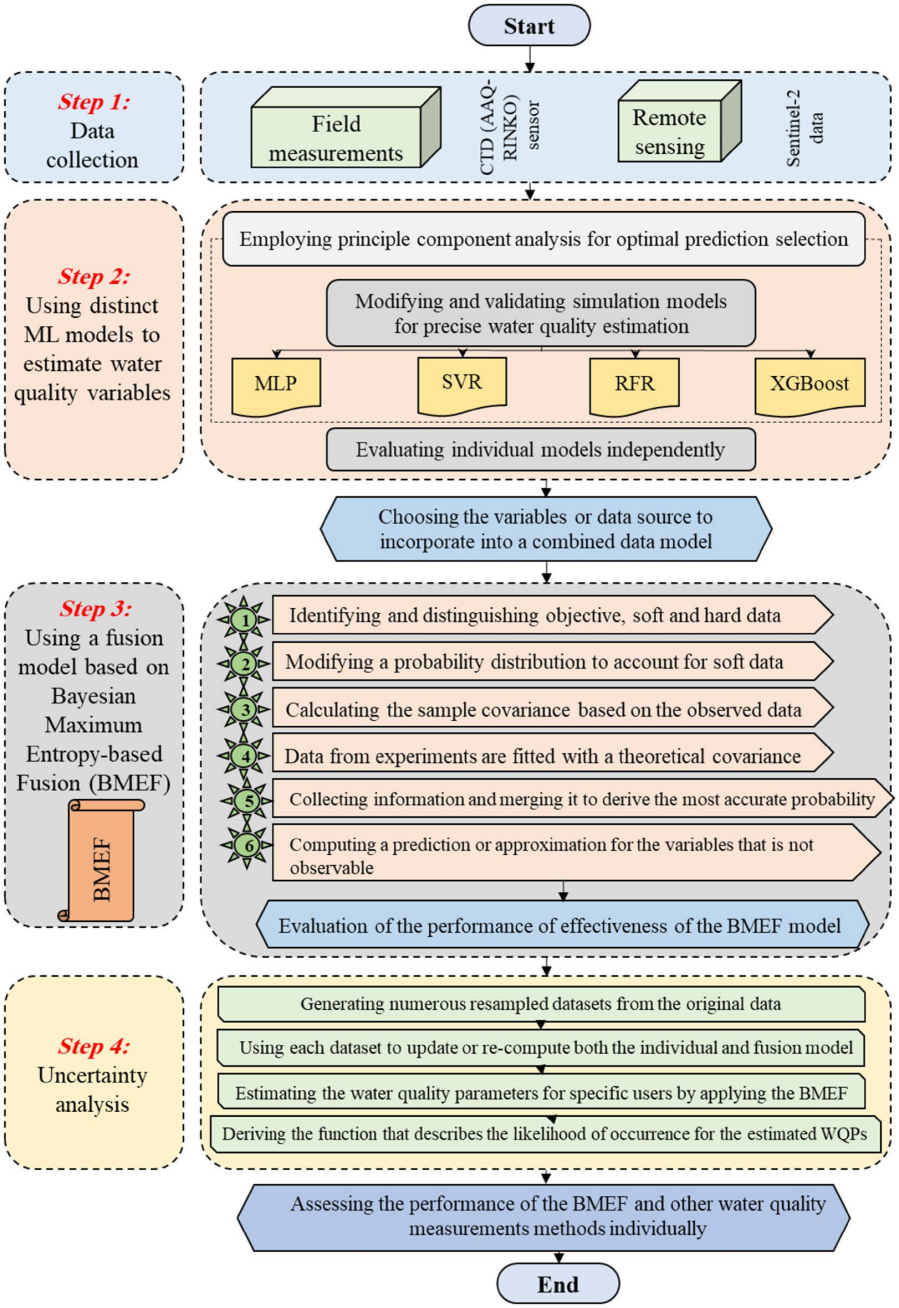
The flowchart of the proposed methodology.

Four types of ML algorithms and the BMEF model were employed to estimate Chl-a and DO to compare the effectiveness of ML algorithms in estimating WQIs using RS data. These characteristics were obtained from an AAQ-RINKO sensor. Figure 1 depicts the flowchart outlining the suggested methodology to estimate WQIs. The structure encompasses the subsequent stages:

1. Data gathering

The data used to establish the ML models was gathered using an AAQ-RINKO sensor for 15th January, 30th January, 14th February, 1st March, 31th March, 20th April, 30th April, 25th May, 31th May, 9th June, 4th July, and 14th July 2023 in 254 sampling points. In addition, as the fundamental purpose of the present research was to develop ML algorithms able to accurately estimate WQIs using S2 data, the value of different bands, namely Aerosols (B1), blue (B2), green (B3), red (B4), Red Edge 1 (B5), Red Edge 2 (B6), Red Edge 3 (B7), NIR (B8), water vapor (B9), Cirrus (B10), shortwave infrared-1 (B11), and shortwave infrared-2 (B12), were extracted for the sampling points. It is worth mentioning that the process of atmospheric correction is done based on the SIAC algorithm.

2. Optimizing predictor selection (input variables)

This paper determines the most effective predictors among various variables are B1, B2, B3, B4, B5, B6, B7, B8, B9, B10, B11, and B12, which are selected as models’ inputs for estimating WQIs. To do so, feature selection was performed based on a trial-and-error procedure to find important variables for predicting WQIs, namely DO and Chl-a. Additionally, using Pearson's correlation coefficient, WQIs with the most robust links with various bands of satellite data were obtained to develop ML algorithms.

3. Developing ML algorithms

During this stage of the proposed framework, the WQPs, including ORP, temperature, EC, DO, and Chl-a are estimated individually using the individual models that have been calibrated. The ANN, SVR, RF, and XGBoost were utilized to develop these data-driven forecasting models.

4. Developing a hybrid model

A fusion model rooted in BMEF was created with the aim of improving the results achieved through singular ML algorithms. BMEF heralds a paradigm shift by seamlessly amalgamating diverse learning models through Bayesian inference and maximum entropy. As an innovative fusion method, BMEF catalyzes advancements in computational intelligence, promising a new era of predictive prowess.

5. Comparing the results of Singular and fusion models considering uncertainty

During this phase, the findings of the new procedure, that is, BMEF, and singular models were subjected to uncertainty analysis using a resampling technique and MCA.

### MLR-ANNs

**Figure 2:**
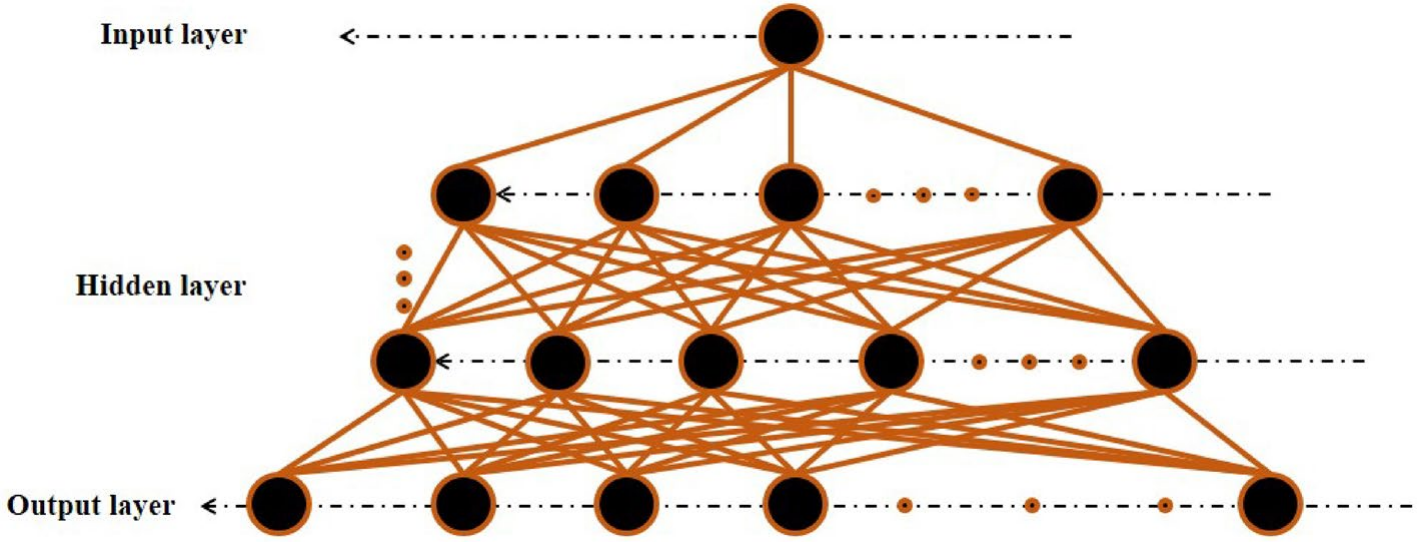
Architecture of the MLR-ANN model in this study.

ANNs are part of the most practical and effective tools for ML algorithms employed in regression problems to estimate output(s) regarding the specific nature of the problem. ANNs, which take inspiration from the human brain, can effectively model various highly intricate nonlinear problems and deliver precise outcomes^63^. This method can prove useful in situations where the data does not conform to established MFs^64^. MLR-ANNs are among the most prevalent and applicable types of ANNs, as depicted in Fig. 2. In addition, *net* and *z* can be computed using the following procedure, as described in Eqs. (1) and (2):

1 $$net = b + \mathop \sum \limits_{i = 1}^{n} W_{i} X_{i}$$

2 $$z = f\left( {net} \right)$$

The variables $X_{1}$ to $X_{n}$ are the first to $n{\text{th}}$ input variables; $W_{1}$–$W_{n}$ represent their respective weights; the variable *b* is a constant; *f* indicates a function called “transfer”; and *z* is the neuron's output. The weights, $W_{i}$, and constant number, b, neurons weights in ANNs are determined through an optimization method. Three transfer functions are frequently used in ANNs, which include the hyperbolic tangent sigmoid (TANSIG), logarithmic sigmoid (LOGSIG), and pure linear transfer function (PURELIN). Equations (3)–(5) represent the three transfer functions used in ANNs.

3 $$TANSIG\left( x \right) = \frac{2}{{1 + e^{ - 2x} }} - 1$$

4 $$LOGSIG\left( x \right) = \frac{1}{{1 + e^{ - x} }}$$

5 $$PURELIN\left( x \right) = x$$

### SVR

**Figure 3:**
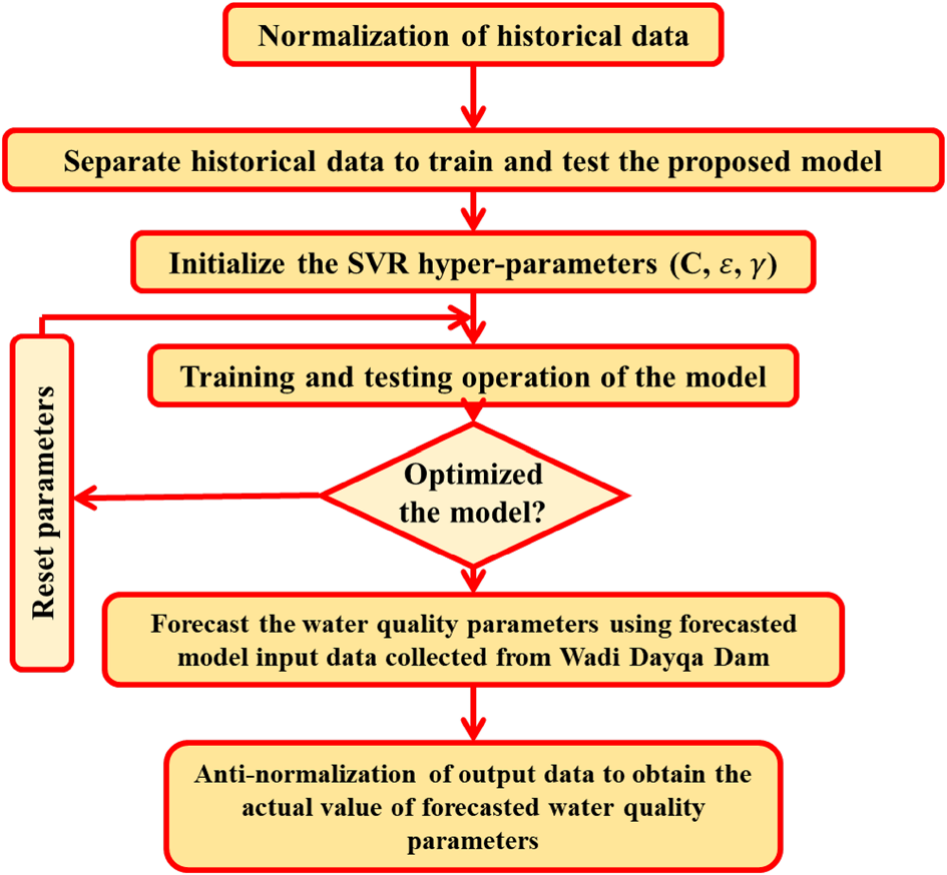
Architecture of the SVR model in this study.

SVR is a traditional supervised ML technique that was initially presented by^65^. SVR is a method even when there is no pre-knowledge of the raw data. This technique can be employed to unstructured or semi-structured data, such as text or images, and it is carried out within the framework of statistical learning theory and minimal structure risk concept^66^. Numerous papers and books provide extensive information on the underlying principles and mathematical formulas of SVR; therefore, this paper does not repeat these equations and the core concept of SVR. Figure 3 illustrates the utilization of the SVR model in estimating WQIs, which includes the latitude, longitude, and depth as inputs, as well as the WQIs as outputs of the developed model.

### RFR

**Figure 4:**
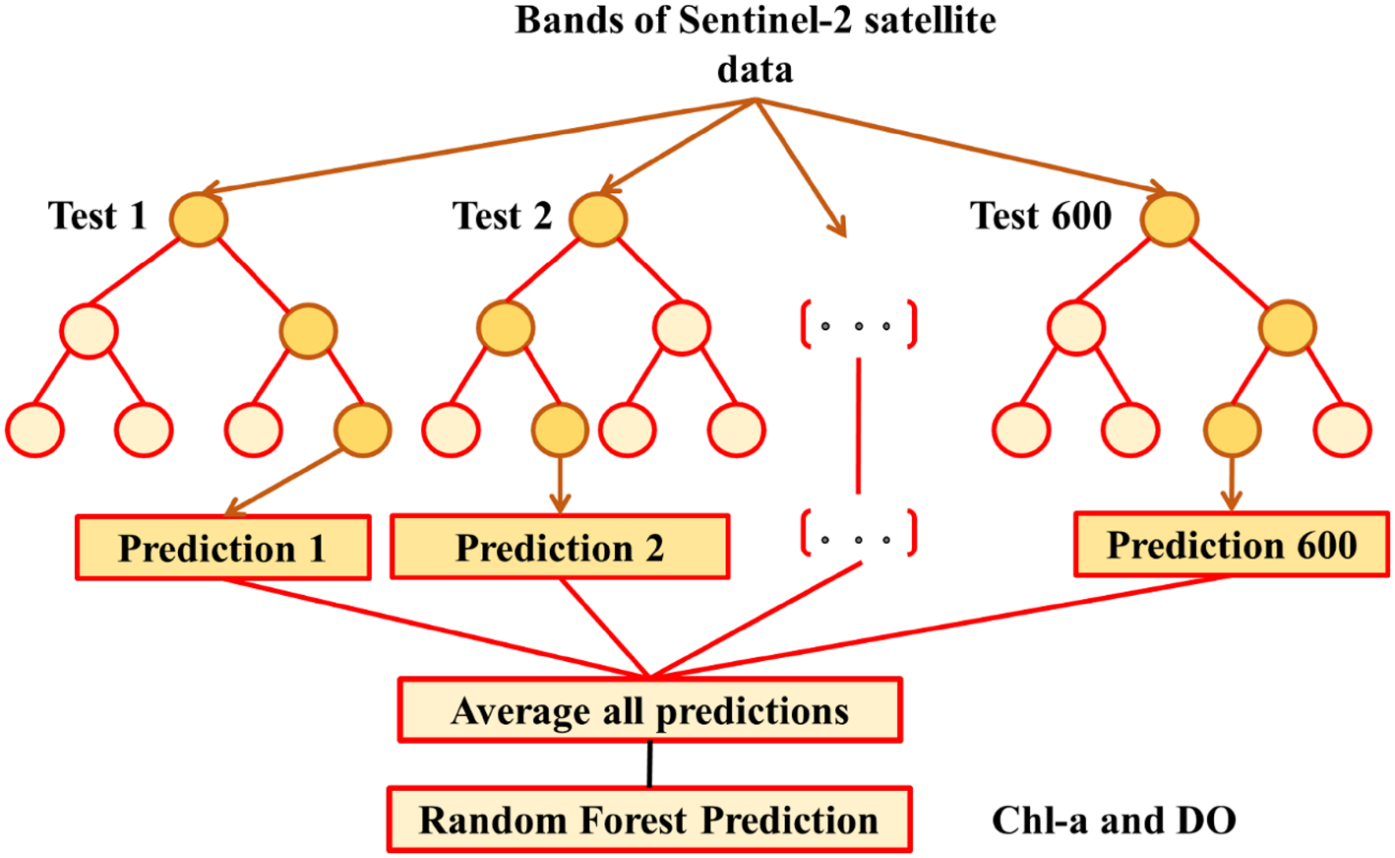
Architecture of the RFR in this study.

The ML approach called RF employs numerous decision trees and a collection of predictors to predict and assess outcomes^67^. RF attains its predictive outcomes by forming decision trees through a training dataset^68^. RF creates multiple decision trees, with each tree autonomously cultivated using a distinct bootstrap sample from the training data (Fig. 4). The bootstrap sampling procedure is utilised to randomly partition the acquired data into homogeneous subsets. The credibility and precision of each tree are ascertained through the assessment of the remaining samples, following its generation and training on the data randomly selected.

During the initial comparison phase, which focused solely on selecting the optimal model, the grid search technique was utilized to enhance the quality of tuning hyperparameters. Therefore, the RFR model was utilized to establish the association between the potential predictors (the value of spectral bands) and the target variables (in-situ WQIs).

### Gradient Boosting Algorithms

**Figure 5:**
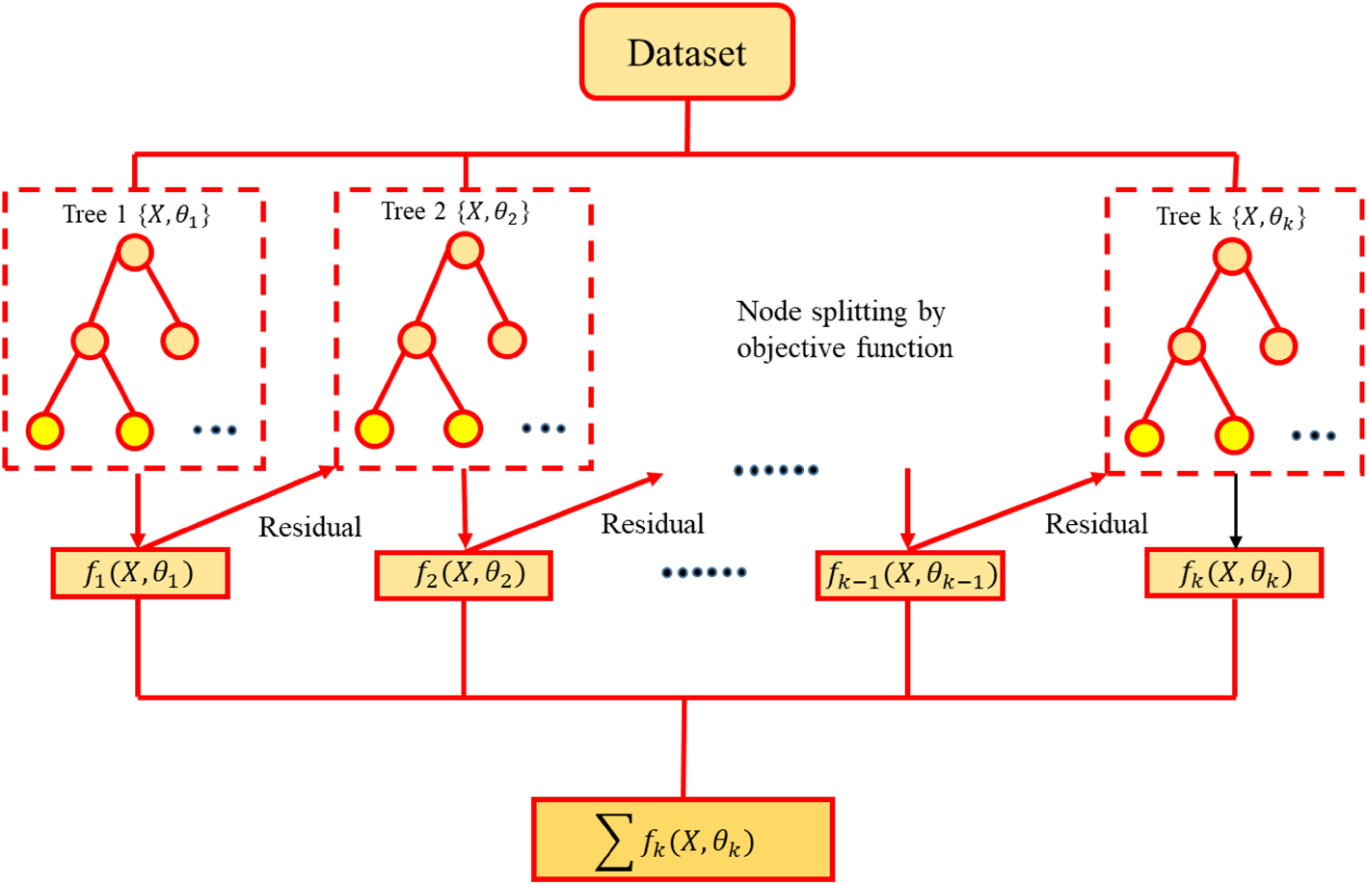
Architecture of the XGBoost model in this study.

A gradient boosting algorithm is a useful tool for predicting a substantial volume of data characterized by exceptional accuracy. This type of algorithm belongs to the boosting family, which combines the predictions of multiple base estimators to enhance accuracy^69^; specifically, the process of consolidating various weak or moderate indicators into robust indicators is known as fusion^70^. A guiding heuristic of boosting is that increasingly refined approximations can lead to good predictive results by consolidating the predictions of weak or normal indicators into more solid ones. XGBoost stands as an optimized distributed gradient boosting library that boasts productivity, adaptability, and convenience as its hallmarks. Additionally, XGBoost is an AI library that performs calculations using the gradient boosting system. Such a system can perform AI computations^71^. It is known for its dominance in structured or tabular datasets when it comes to classification and regression predictive modeling problems. Moreover, it has the ability to solve numerous issues in data science rapidly and with precision. Figure 5 displays the XGBoost architecture considered for water quality prediction in this study.

### CV approach for ML algorithms

CV offers a means of evaluating a model’s predictive capacity on new and unseen data, yielding crucial insights into the model’s ability to generalize beyond its training set. It furnishes an estimate of the model’s potential performance when applied to unfamiliar data points^72^. One strategy to address concern involves abstaining from using the entire dataset during the training phase. Before initiating the training process, the remaining data is segregated and kept separate. Upon concluding the training phase of the dataset, the data that remains is employed to evaluate the proficiency of the algorithms. Such principle constitutes the foundational idea underlying an extensive range of model evaluation methodologies referred to as CV.

**Figure 6:**
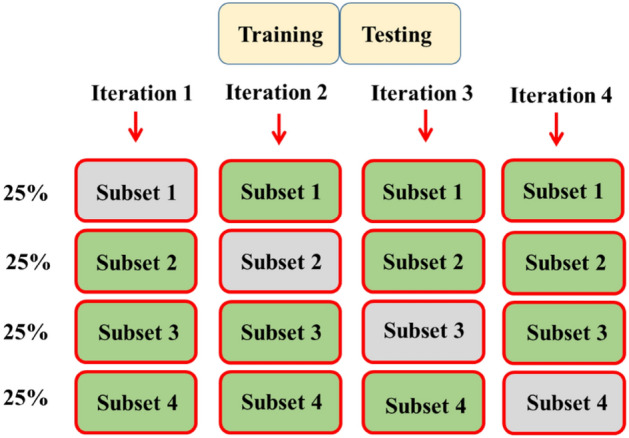
The utilization of CV methodology.

The portioning datasets hold the capability to influence the results of ML algorithms. Numerous methods to develop the idea of employing CV have been put forth in prior research endeavors. Nonetheless, the fundamental elements of all these methodologies share a common essence^71^. The hold-out procedure was selected for this study due to its uncomplicated and straightforward nature among the assortment of available techniques. In the present study, Fig. 6 illustrates the CV methodology employed.

### BMEF model

In this study, the capabilities of the geospatial approach called BME were utilized. In addition, a fusion model based on the BME principle was employed by combining the outcomes with various estimation techniques. BMEF stands as a geostatistics-derived technique capable of amalgamating variables or spatially extrapolating them across multiple locations when data is lacking. This approach achieves equilibrium between two scenarios: (1) incorporating prior knowledge and familiarity with the spatial fluctuations of estimated variables characterized by maximum entropy and (2) pursuing the optimization of Bayesian functions to attain posterior probabilities with minimal uncertainty.

**Figure 7:**
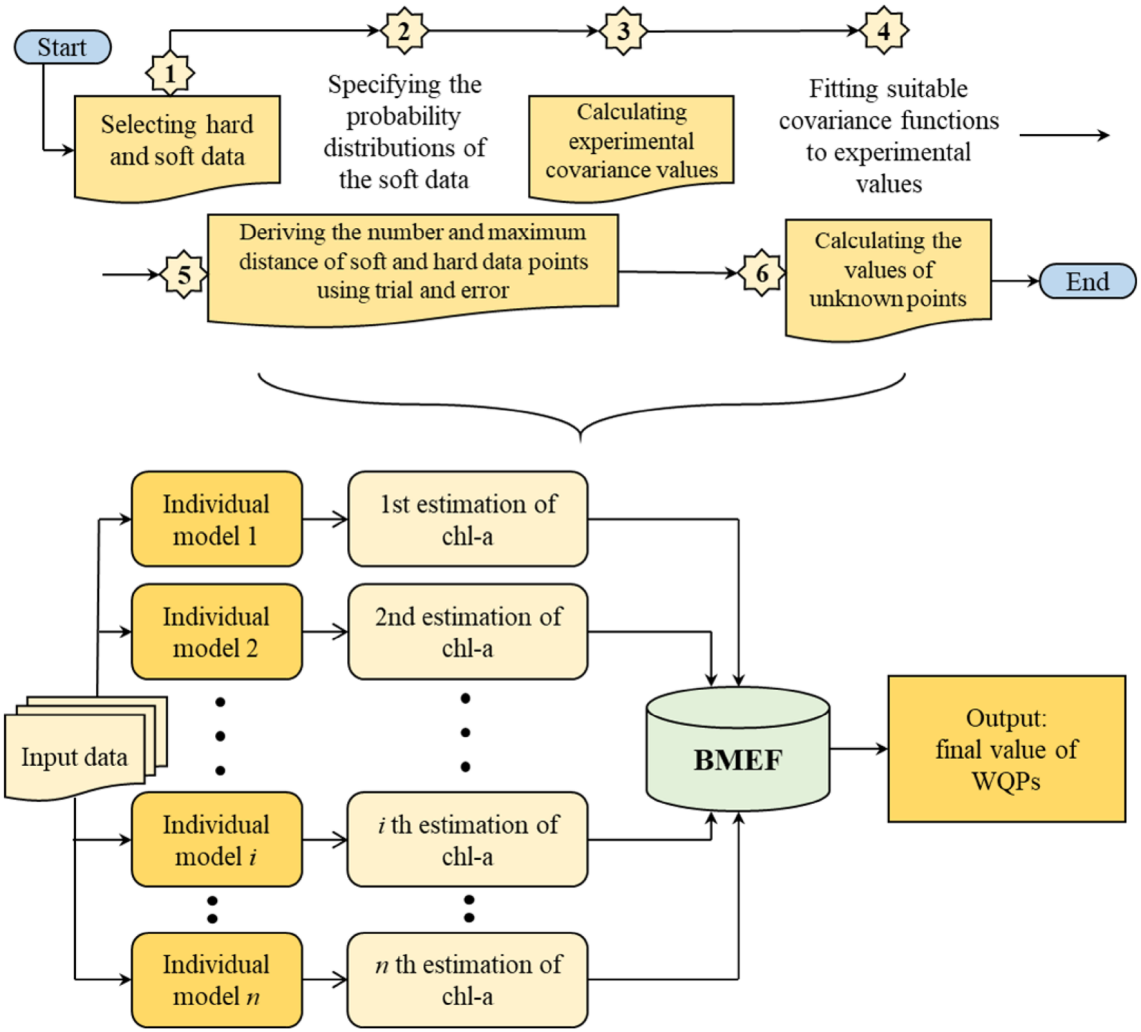
A schematic of fusion steps using the BMEF model.

Within this study, we contemplate a series encompassing the 1st through nth predictions. This sequence serves as a depiction of projected WQIs generated by employing three distinct models: MLR, RFR, and SVR. The BMEF model considers the predictions of the less accurate values for the parameters of the water quality made using the three different models^73^. Considering the fact that BMEF implements the distinctive benefit offered by each model, the newly devised model demonstrates enhanced assurance and precision in fulfilling the WQIs. The diagram illustrating the amalgamation methodologies that utilize the developed BMEF model is illustrated in Fig. 7. Furthermore, the steps involved in BMEF are explained in detail below:

1. Introducing spatiotemporal data of estimates into *n* separate trained and tested model.
2. Employing distinct algorithms to generate preliminary estimates for WQIs.
3. WQIs are considered vectors computed through singular estimation models, and the pertinent observation is associated with them. In relation to the symbol representing this vector, ($x_{1j} , x_{2j} , \ldots , x_{nj} , z_{j}$), where, $x_{1} , x_{2} , \ldots , x_{n}$ are 1st to nth estimation of the factors influencing WQIs generated by each ML model across different depths ranging from *i* to *n*, and ${z}_{j}$ represents the recorded WQIs at a determined depth of the reservoir.
4. Determining the measure of the distinction between singular data points and their corresponding observations: as a model’s predictions align more closely with the corresponding data, its relative influence increases within the fusion process.
5. Selecting types of data, that is, soft and hard, regarding the outcomes derived from ML and recorded data. The foremost advantage provided by the BMEF over earlier data fusion models lies in its ability to incorporate uncertainty into the problem as a probabilistic number. By partitioning the data into two categories, the present data uncertainties are taken into account during the projection of WQIs. The former data can manifest as range values or PMF and frequently encompass estimation as well as observational inaccuracies. Thus, the WQIs produced from singular ML models are considered “soft data.” Consequently, rather than employing an exact numerical value, we may include the distinct models’ estimations with a larger level of uncertainty. To capture the characteristics of soft data, we use ML algorithms to compute PMFs.
6. Computing the covariance values obtained through experimentation: The BMEF analysis establishes how the distances between the data points related to their importance. In this context, the term 'data points' refers to the estimated values of WQIs. The space separating such data points reflects the variations among the calculated water quality metrics from different models and their corresponding actual values. Covariance is a determined type of statistical correlation function that indicates this importance. As a result, experimental variogram points can be developed to demonstrate the correlation between covariance values and distances.
7. Creating appropriate variograms that align with the empirical values: The empirical samples are aligned with a designated MF referred to as a theoretical variogram. In this study, the observed locations were used to fit a spatial covariance model using the hard data. This generated graph was employed to calculate the probability distribution function (PDF) of WQIs.
8. Employing the provided variogram to estimate values for undetermined samples.

### Assessing the effectiveness of ML techniques

Several statistical indicators can be used to assess the precision and margin of error exhibited by the models that were developed. In the current study, such indicators as Nash–Sutcliffe (NSC), mean squared error (MSE), mean absolute error (MAE), root mean square error (RMSE), and R-squared ($R^{2}$) were employed to assess the accuracy and error of each model^91^. These indices are defined in Eqs. (6)–(10)^74^:

6 $$NSC = 1 - \frac{{\mathop \sum \nolimits_{i = 1}^{n} \left( {T_{i} - P_{i} } \right)^{2} }}{{\mathop \sum \nolimits_{i = 1}^{n} \left( {T_{i} - \overline{P}} \right)^{2} }}$$

7 $$MAE = \frac{1}{n}\mathop \sum \limits_{i = 1}^{n} \left( {\left| {T_{i} - \overline{T}_{i} } \right|} \right)$$

8 $$MSE = \frac{{\mathop \sum \nolimits_{i = 1}^{n} \left( {T_{i} - P_{i} } \right)^{2} }}{n}$$

9 $$RMSE = \sqrt[2]{{\frac{{\mathop \sum \nolimits_{i = 1}^{n} \left( {T_{i} - P_{i} } \right)^{2} }}{n}}}$$

10 $$R^{2} = \left( {\frac{{\left( {T_{i} - \overline{T}} \right) - \left( {P_{i} - \overline{P}} \right)}}{{\sqrt {\mathop \sum \nolimits_{i = 1}^{n} \left( {T_{i} - \overline{T}} \right)} \mathop \sum \nolimits_{i = 1}^{n} \left( {P_{i} - \overline{P}} \right)}}} \right)^{2}$$

wherein *T* represents the desired amount; *P* represents the estimated value; $\overline{T}$ represents the mean value of the target data; $\overline{P}$ represents the mean of the data forecasted by the model; and *n* represents the count of data analyzed. Each of the aforementioned statistical indicators was computed independently.

### Spectral-band importance

RS for WQI estimation has two key factors. The first is the algorithm used to detect the relationship between reflectance from different spectral bands of the satellites, and the second is the band selection for every parameter. Different ML algorithms and other statistical algorithms, such as regression, have been used to find the relation between satellite bands’ reflectance and field measurements. This study conducted a variable-importance analysis to assess the effect of different wavelengths or spectral bands on ML models. The analysis employed various band reflectance values as input features to estimate the concentration of the corresponding water-quality variable. By doing so, the study aimed to determine which wavelengths or spectral bands have the most significant impact on ML algorithms. Overall, remote sensing technology offers a powerful tool for assessing and monitoring WQIs over large spatial scales, facilitating informed decision-making and sustainable management of aquatic resources.

## Case study

### Study area

Oman, characterized as a semi-arid nation, possesses limited reserves of enduring surface water. Among the scarce wadis in Oman maintaining consistent flow, Wadi Dayqah stands out^75^. Surrounding this reservoir are diverse mountains, an array of wadis, and fertile grounds, collectively rendering the area an attractive destination. The WDD demonstrates the promise of hydropower technology and possesses immeasurable economic significance^89^. Climate and precipitation transitions occur from summer to winter, spanning from mountainous regions to coastal areas or wadis. Unlike the mountainous area, the lowland sections of the reservoir encounter elevated temperatures and humidity levels^90^. Furthermore, winter brings precipitation patterns, with certain years experiencing notably higher rainfall, leading to substantial floods due to the presence of other wadis in close proximity to the dam. The area's consistent radiation throughout the year and prevailing arid climate contribute to a relatively modest flow of water through the wadis and wells.

**Figure 8:**
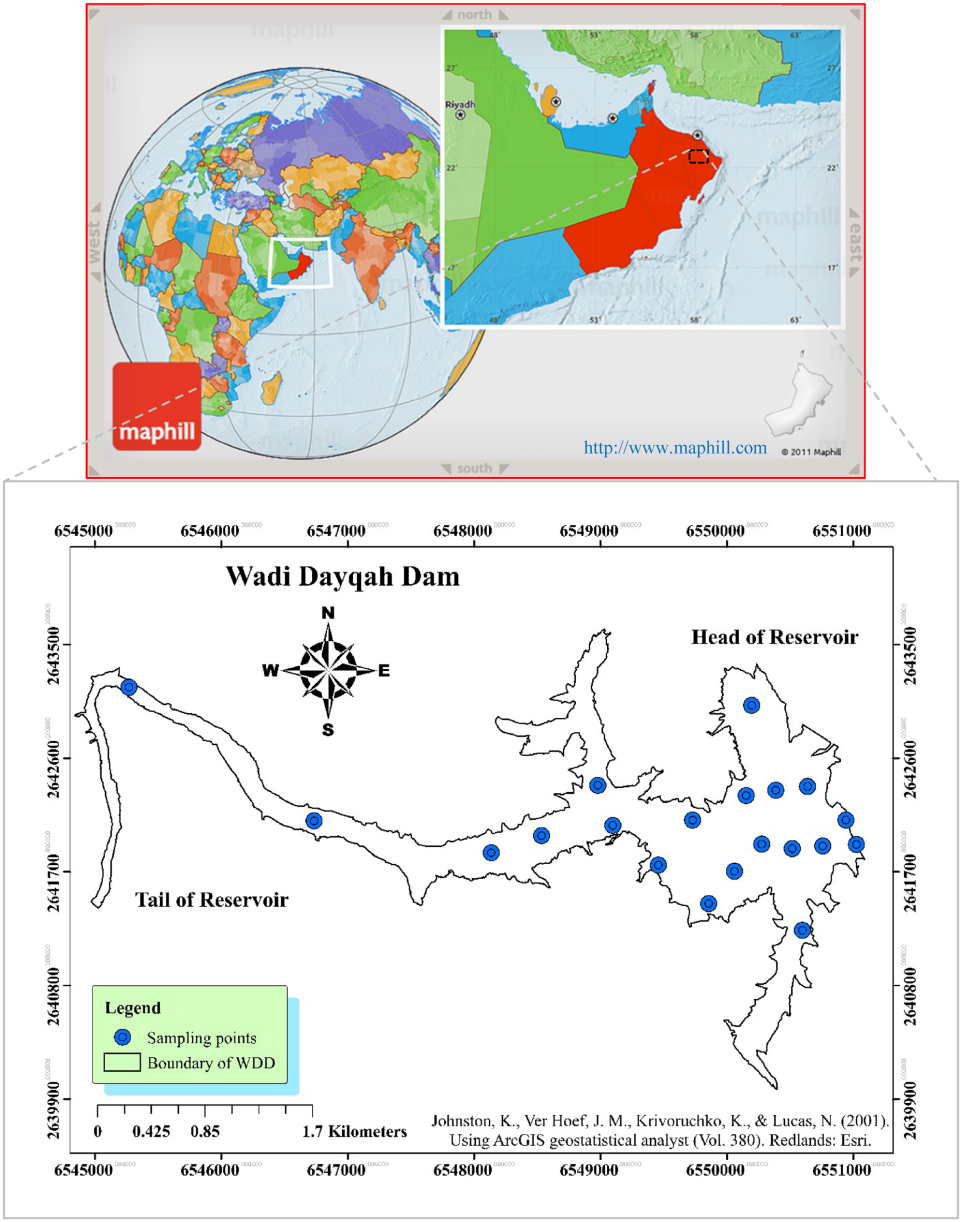
Location of Wadi Dayqah Dam and its designated sampling points, developed by ArcMap^76^.

Thermal stratification refers to temperature variations at various reservoir depths, influencing the density of the water. Prolonged water retention periods within this reservoir, coupled with thermal stratification, lead to (1) notable deterioration in water quality; (2) an elevated likelihood of water contamination, posing risks to human health; and (3) heightened treatment demands for the WDD. Significant to recognize is that stratification divides the reservoir water into distinct layers, with the lower layer lacking atmospheric oxygen. Figure 8 depicts the perimeter of the WDD along with the sampling stations designated for acquiring WQIs.

### Data

This study utilized data from an in situ spectroscopy and water-quality measurements collected through a CTD sensor. The CTD sensor, an acronym for conductivity, temperature, and depth sensor, serves as a fundamental instrument in oceanographic and limnological research. This sensor enables precise measurement of critical water quality indicators, including salinity, temperature, and pressure, thereby facilitating comprehensive understanding and real-time monitoring of aquatic environments. Table 1 summarizes the WQIs collected via the CTD sensor. The researchers conducted field sampling from January 15th, 2023 to July 14th, 2023, to collect water quality data. A total of 254 samples were collected, including temperature, EC, pH, Chl-a, ORP, and DO. In addition, field spectroscopy data were collected using S2. The S2 mission offers high-resolution, multi-spectral images that can be utilized in various fields of study. The S2 Multi-spectral Imagery (MSI) data consists of 13 bands. Table 2 Provides detailed information on the S2 bands. The satellite images of four dates in 2023 (15 January, 30 January, 14 February, 1 March, 31 March, 20 April, 30 April, 25 May, 31 May, 9 June, 4 July, and 14 July) were downloaded using Google Earth Engine (GEE). All of the computational processes on satellite images were done utilizing GEE. This paper utilized a total of 254 datasets, of which 190 were used for training and calibration purposes, and the remaining 64 datasets were used to verify the developed models.

**Table 1:** Statistical summary of recorded WQIs in WDD.

WQPs	Mean	Variance	Standard deviation	Minimum	Maximum	Skewness	Kurtosis
Temperature	23.27	0.25	0.50	22.045	24.338	− 0.528	− 0.097
EC	565.53	0.25	8.43	555.4	610.6	2.10	8.07
Chl-a	0.71	0.18	0.43	0.117	2.858	2.85	10.94
pH	7.55	0.17	0.41	4.91	8.62	− 2.68	17.52
ORP	101.12	4592	67.78	− 31.12	300.77	0.55	0.08
DO	7.69	0.12	0.35	6.98	8.37	− 0.25	− 0.86

The statistics are computed from 254 samples.

**Table 2:** Summary of various bands of Sentinel-2.

Name	Resolution	Wavelength	Description
B1	60 m	443.9 nm (S2A)/442.3 nm (S2B)	Aerosols
B2	10 m	496.6 nm (S2A)/492.1 nm (S2B)	Blue
B3	10 m	560 nm (S2A)/559 nm (S2B)	Green
B4	10 m	664.5 nm (S2A)/665 nm (S2B)	Red
B5	20 m	703.9 nm (S2A)/703.8 nm (S2B)	Red Edge 1
B6	20 m	740.2 nm (S2A)/739.1 nm (S2B)	Red Edge 2
B7	20 m	782.5 nm (S2A)/779.1 nm (S2B)	Red Edge 3
B8	10 m	835.1 nm (S2A)/833 nm (S2B)	Near-Infrared (NIR)
B8A	10 m	864.8 nm (S2A)/864 nm (S2B)	Red Edge 4
B9	20 m	945 nm (S2A)/943.2 nm (S2B)	Water vapor
B11	20 m	1613.7 nm (S2A)/1610.4 nm (S2B)	SWIR 1
B12	20 m	2202.4 nm (S2A)/2185.7 nm (S2B)	SWIR 2

### Uncertainty analysis

**Figure 9:**
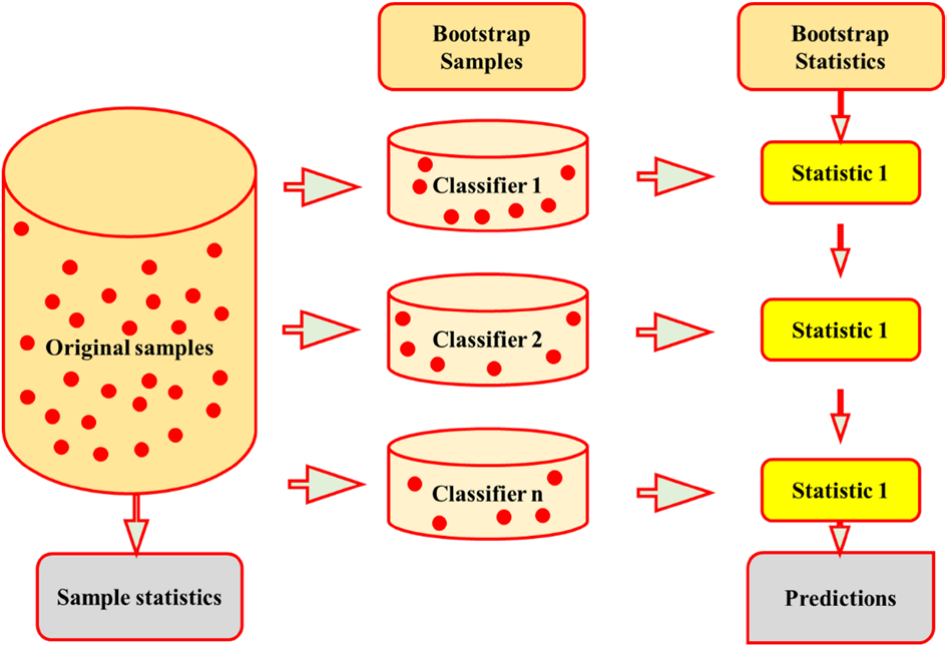
A flowchart of the bootstrap resampling method.

In this study, resampling and MCA are used to analyze uncertainties on both the singular outcomes of estimated algorithms and the BMEF (Fig. 9). The bootstrap approach, which is a resampling and introduced by^77^ methodology, is used to calculate the statistical characteristics of a variable. The foundation of this approach rests on the premise that when information about the proper distribution of a variable is lacking, sampled values offer the most accurate estimation. Additionally, an adequate estimate of the distribution of the unfamiliar population is possible using the established practical distribution obtained through a recorded dataset. Such a procedure proves valuable for analysis due to its relative ease of use in contrast to conventional analytical approximations, e.g.,^77^. According to^78^, the bootstrapping strategy is built upon two key components: unbiased drawing samples by drawing with the substitution from the initial dataset to develop an MC approximation of the bootstrap resampling technique.

The bootstrapping process comprises two main phases: conducting autonomous and random sampling with replacement from the primary dataset to acquire a Monte Carlo approximation of the bootstrap resampling approach. More details about this technique can be found in^78^.

## Results and discussions

In the current research, four singular ML techniques and the BMEF model, a combination of these techniques, were formulated to augment the outcomes yielded by standalone ML algorithms. Field-measured data for each parameter was the dependent variable, and the S2 reflectance for each band at the same location of the field-measured data was the model predictor. Model validation was performed using 25% of the data for testing and 30% for the training dataset. The decision to utilize a 30% training dataset was meticulously reasoned, considering both the intricacies of the dataset and the computational constraints, ensuring that the selected machine learning algorithms demonstrate robust performance and generalize effectively to unseen data despite the relatively smaller training size.

### ML models’ inputs and outputs

As the main goal of this research was to map WQPs using different bands of satellite datasets, the sampling WQIs obtained from a CTD sensor were considered target values. Furthermore, the value of various bands of S2 was considered as the input variable or feature of ML algorithms. RS for WQIs estimation has two key factors: (1) the algorithm used to detect the relationship between reflectance from different spectral bands of the satellite; and (2) the band selection for every parameter. Different ML algorithms and other statistical algorithms, such as regression, were used to find the relation between satellite bands’ reflectance and field measurements. Four different date field measurements, having the same time as S2, have been obtained to recognize the best selection of bands. The final surface water quality of each parameter was exported for the best band combination.

### Fine-tuning the ML algorithms

The tuning mechanism entails identifying the optimal hyperparameter for a learning algorithm pertaining to distinct data. In the realm of supervised learning, the term 'optimally' encompasses various performance indicators and run time, which hyper-parameters can significantly impact in some cases. The enhancement in performance achievable by modifying a single hyper-parameter compared to its preset configurations is known as ''tunability,'' encompassing all hyper-parameters. In order to proceed with the process of tuning in the current research, the optimal proportion of four ML procedures, namely MLR, RFR, SVR, and XGBoost, were obtained via trial and error.

The MLR algorithm's development in Keras involved utilizing an MLR. As was discussed, the parameters of this algorithm were tuned via a trial-and-error procedure. The model considered in this study comprised an input layer that represented the different bands of S2 satellite data. Considering more details, the first hidden layer consisted of fifty neurons, while the second contained twenty-five. The optimization approach to minimize the MAE quantity was determined based on Adam. This optimization technique choice aligns with utilizing the ReLU activation function in both the hidden layers and the output layer. Table 3 provides an overview of the HPOs in the MLR model.

**Table 3:** The summary of optimized parameters obtained for machine learning algorithms.

Model	Predicted Variable	Structure
MLP	Temperature	# layers: 3; # Nodes: 20; HAF: ReLU; RAF: Sigmoid; OAF: ReLU; Dropout: 0.001; RD: 0.05; LF: MAE; OA: Adam
	EC	# layers: 3; # Nodes: 25; HAF: ReLU; RAF: Sigmoid; OAF: ReLU; Dropout: 0.001; RD: 0.05; LF: MAE; OA: Adam
	Chl-a	# layers: 3; # Nodes: 20; HAF: ReLU; RAF: Sigmoid; OAF: ReLU; Dropout: 0.001; RD: 0.05; LF: MAE; OA: Adam
	pH	# layers: 3; # Nodes: 25; HAF: ReLU; RAF: Sigmoid; OAF: ReLU; Dropout: 0.001; RD: 0.05; LF: MAE; OA: Adam
	ORP	# layers: 3; # Nodes: 20; HAF: ReLU; RAF: Sigmoid; OAF: ReLU; Dropout: 0.001; RD: 0.05; LF: MAE; OA: Adam
	DO	# layers: 3; # Nodes: 25; HAF: ReLU; RAF: Sigmoid; OAF: ReLU; Dropout: 0.001; RD: 0.05; LF: MAE; OA: Adam
SVR	Temperature	Kernel: Linear kernel; Sigma: 1.0; Epsilon: 0.1; C: ; and degree of the polynomial: -
	EC	Kernel: Polynomial; Sigma: 1.0; Epsilon: 0.1; C: 1.0; and degree of the polynomial: 3
	Chl-a	Kernel: RBF kernel; Sigma: 1.2; Epsilon: 0.1; C: 1.0; and degree of the polynomial: -
	pH	Kernel: Linear kernel; Sigma: 1.04; Epsilon: 0.17; C: 1.0; and degree of the polynomial: -
	ORP	Kernel: Polynomial; Sigma: 1.1; Epsilon: 0.15; C: 1.0; and degree of the polynomial: 3
	DO	Kernel: RBF kernel; Sigma: 1.12; Epsilon: 0.15; C: 1.0; and degree of the polynomial: -
RFR	Temperature	Max_depth: 15; Criterion: MSE; Min_size: 7; n_trees: 1000; n_features: 2
	EC	Max_depth: 15; Criterion: MSE; Min_size: 7; n_trees: 1000; n_features: 2
	Chl-a	Max_depth: 15; Criterion: MSE; Min_size: 7; n_trees: 1000; n_features: 2
	pH	Max_depth: 15; Criterion: MSE; Min_size: 7; n_trees: 1000; n_features: 2
	ORP	Max_depth: 15; Criterion: MSE; Min_size: 7; n_trees: 1000; n_features: 2
	DO	Max_depth: 15; Criterion: MSE; Min_size: 7; n_trees: 1000; n_features: 2
XGBoost	Temperature	Learning_rate = 0.1; n_estimator = 100; subsample = 1.0; criterion = ‘friedman’; max_depth = 5, ccp_alpha = 0
	EC	Learning_rate = 0.1; n_estimator = 100; subsample = 1.0; criterion = ‘friedman’; max_depth = 5, ccp_alpha = 0
	Chl-a	Learning_rate = 0.1; n_estimator = 100; subsample = 1.0; criterion = ‘friedman’; max_depth = 5, ccp_alpha = 0
	pH	Learning_rate = 0.1; n_estimator = 100; subsample = 1.0; criterion = ‘friedman’; max_depth = 5, ccp_alpha = 0
	ORP	Learning_rate = 0.1; n_estimator = 100; subsample = 1.0; criterion = ‘friedman’; max_depth = 5, ccp_alpha = 0
	DO	Learning_rate = 0.1; n_estimator = 100; subsample = 1.0; criterion = ‘friedman’; max_depth = 5, ccp_alpha = 0

#: Number; HAF, hidden activation function; LF, loss; OA, Optimization algorithm; RAF, recurrent activation function; OAF: output activation function; RD, recurrent dropout.

The RFR algorithm requires the user to set various parameters, namely, the number of observations randomly drawn, the number of variables chosen for every division, the criterion for portioning, the smallest count of instances, and the number of trees. In practice, RF users may be uncertain if adjusting the tuning parameters to different values could improve performance in contrast to the defaults. The tuned hyper-parameters are described in Table 3. One of the primary challenges in enhancing the predictive accuracy of the SVR model is hyper-parameter tuning. Therefore, this study optimized five parameters of the SVR algorithm via trial and error. The SVR model requires various parameters to be set, including (I) the type of kernel function, which can be polynomial, linear, or radial basis functions; (II) sigma; (III) epsilon; (IV) C; and (V) the polynomial function's degree, of the chosen optimal kernel function happens to be of polynomial nature. Figure 3 shows the procedure used for experimenting with various parameter values for the SVR model. Additionally, Table 3 displays the optimal value of each parameter for the SVR model formulated within this research.

#### Outcomes from the utilization of BMEF to estimate WQIs

**Figure 10:**
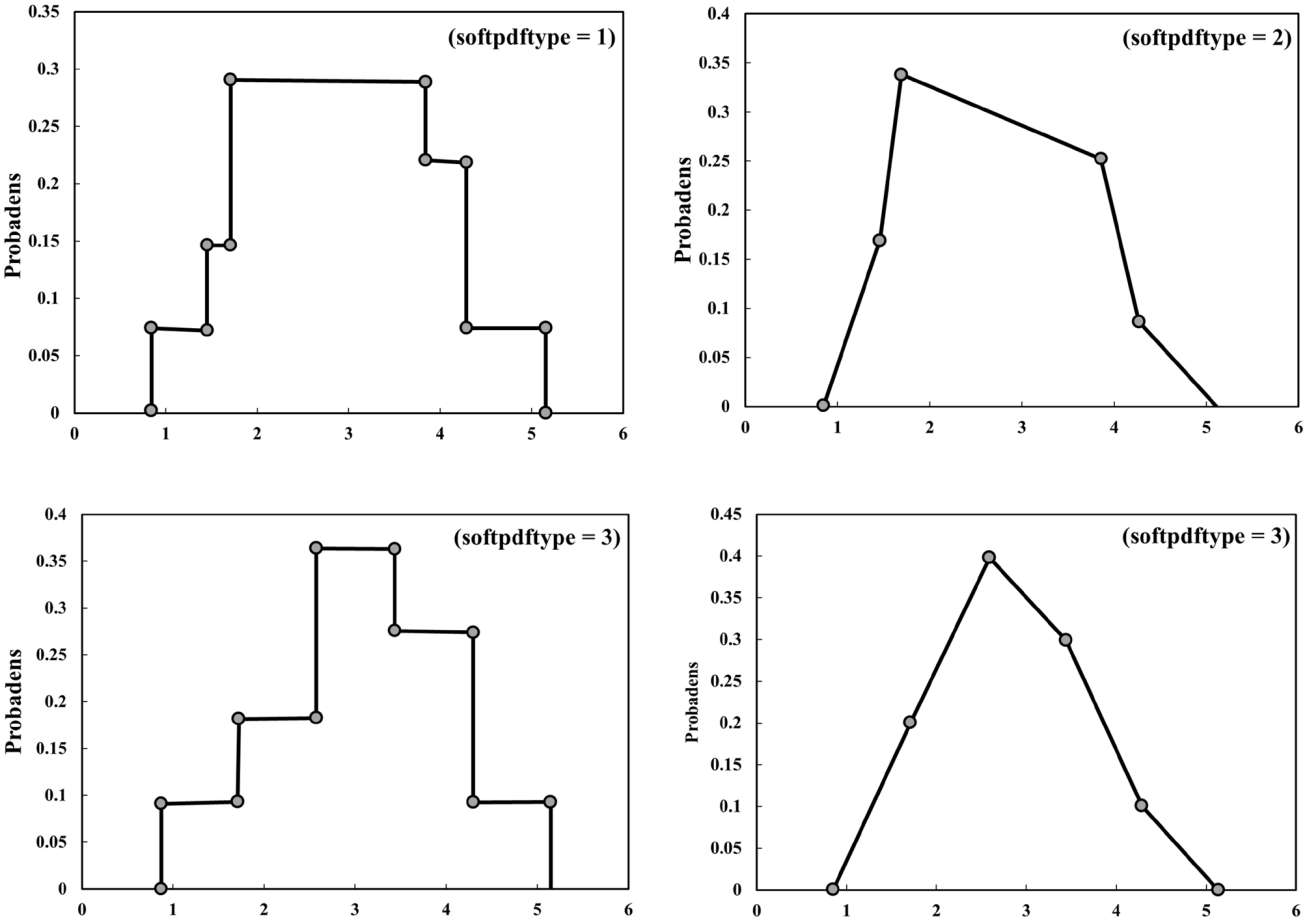
Four main types of probability distribution used in BMEF.

In this part, the results of water quality estimations are presented. These results are based on the utilization of the BMEF and encompass two primary scenarios—all bands of S2 as input variables and those bands having the highest impact and correlation with each of the parameters pertaining to water quality. In order to attain the values of the empirical covariance function, we utilize both recorded and estimated values obtained from singular ML algorithms. Using the MATLAB “cftools” package, the empirical points were employed to conform the appropriate covariance function, and unidentified water quality indices were interpolated using the obtained covariance functions to estimate such parameters. In both scenarios, the estimation of each developed ML algorithm is characterized by elevated uncertainty levels, often referred to as soft data. This method aids in addressing the uncertainty linked to the estimated WQIs characterized by lower precision. Empirical histograms offer a form of characterization for this category of soft data. The main focus of the discussion revolves around four distinct varieties of soft data (Fig. 10) to match the soft data in this study. Furthermore, the probability distribution of type 1 was selected based on the proportionate occurrence rate of the recorded data.

The different models whose outputs are employed in the amalgamation process are shown in Table 4. In addition, Table 4 presents the estimated WQIs using the new methodology of the weighting model. According to the findings obtained from BMEF, employing all bands as input variables as predictor reduced the effectiveness of WQI estimation using RS satellite data because of the limited extent of correlation between some bands and the WQIs. Moreover, the same or better results are typically obtained by considering those bands having a high correlation with WQIs. Section “Evaluation of model performance” discusses the findings of each singular algorithm and the BMEF model in more detail. In the fusion process, four ML algorithms with high accuracy in estimating WQIs are considered predictors in the fusion model.

**Table 4:** The chosen individual algorithms and their outcomes used in the fusion model.

Scenario	Predictor	WQI	Selected individual models		
All bands of S2	B1, …, B12	Chl-a	MLR	RF	SVR
The best combination	B2, B3, B4, B5, B6		MLR	RFR	SVR
All bands of S2	B1, …, B12	DO	MLR	RFR	SVR
The best combination	B2, B3, B4, B8		MLR	RFR	SVR

### Evaluation of model performance

#### The comparison of the standalone ML algorithms as well as the BMEF model

##### Considering S2 bands as input variables

In order to develop ML algorithms, the raw data was separated for the training (70%) and the testing (30%). Subsequently, the ML algorithms and BMEF model, which are mentioned in the previous sections, were developed to estimate the number of WQIs such as DO and Chl-a. Table 5 presents the results of ML and BMEF models, considering all S2 bands as input variables. As can be seen from Table 5, due to the combining merits of individual ML algorithms, the fusion model based on BMEF outperformed all of these developed models. For example, the closer the value of RMSE is to zero, the better results are obtained. These values showed that BMEF leads to better results compared to MLR (15%), RFR (20%), XGBoost (31%), and SVR (19%), respectively. Additionally, considering NSE as an evaluation index, BMEF outperformed other individual ML algorithms by MLR (5%), RFR (2%), XGBoost (6%), and SVR (4%), respectively. It is worth mentioning that the optimum value of NSE is close to one. Based on the results, individual ML algorithms excel in their specialized domains, harnessing specific patterns within data to make estimations. However, they may be limited by their inability to capture the full spectrum of complexity inherent in real-world datasets. The BMEF model, on the other hand, leverages the strengths of multiple individual algorithms, combining their estimation capabilities to develop a more robust and accurate model. By integrating diverse perspectives and insights from various algorithms, the BMEF model outperforms its individual counterparts, offering enhanced estimation power and adaptability across a wider range of scenarios. Through this amalgamation of approaches, fusion models represent a promising frontier in ML, poised to revolutionize estimative analytics across domains.

**Table 5:** The capability of singular ML algorithms considering all bands of S2 as input variables.

Variables	Model	NSE (train)	NSE (test)	$R^{2}$ (train)	$R^{2}$ **(test)**	RMSE (train)	RMSE (test)	MAE (train)	MAE (test)	MSE (train)	MSE (train)
Chl-a	MLR	0.84	0.85	0.86	0.87	2.13	2.17	2.44	2.45	4.53	4.21
	RFR	0.91	0.92	0.88	0.90	2.29	2.31	1.81	1.79	5.24	5.12
	XGBoost	0.82	0.84	0.81	0.81	2.69	2.67	2.78	2.71	7.23	6.95
	SVR	0.85	0.86	0.85	0.86	2.25	2.24	2.23	2.16	5.06	4.14
	BMEF	0.89	0.90	0.91	0.92	1.88	1.85	1.85	1.77	3.53	3.25
DO	MLR	0.84	0.83	0.88	0.89	1.24	1.25	1.48	1.45	1.53	1.43
	RFR	0.81	0.80	0.83	0.84	2.19	2.24	3.25	3.21	4.79	4.61
	XGBoost	0.83	0.82	0.79	0.80	2.24	2.38	4.18	4.11	5.01	5.56
	SVR	0.83	0.84	0.87	0.88	1.28	1.25	0.99	0.97	1.63	1.38
	BMEF	0.91	0.90	0.92	0.91	0.98	0.95	0.83	0.85	0.96	0.82

**Figure 11:**
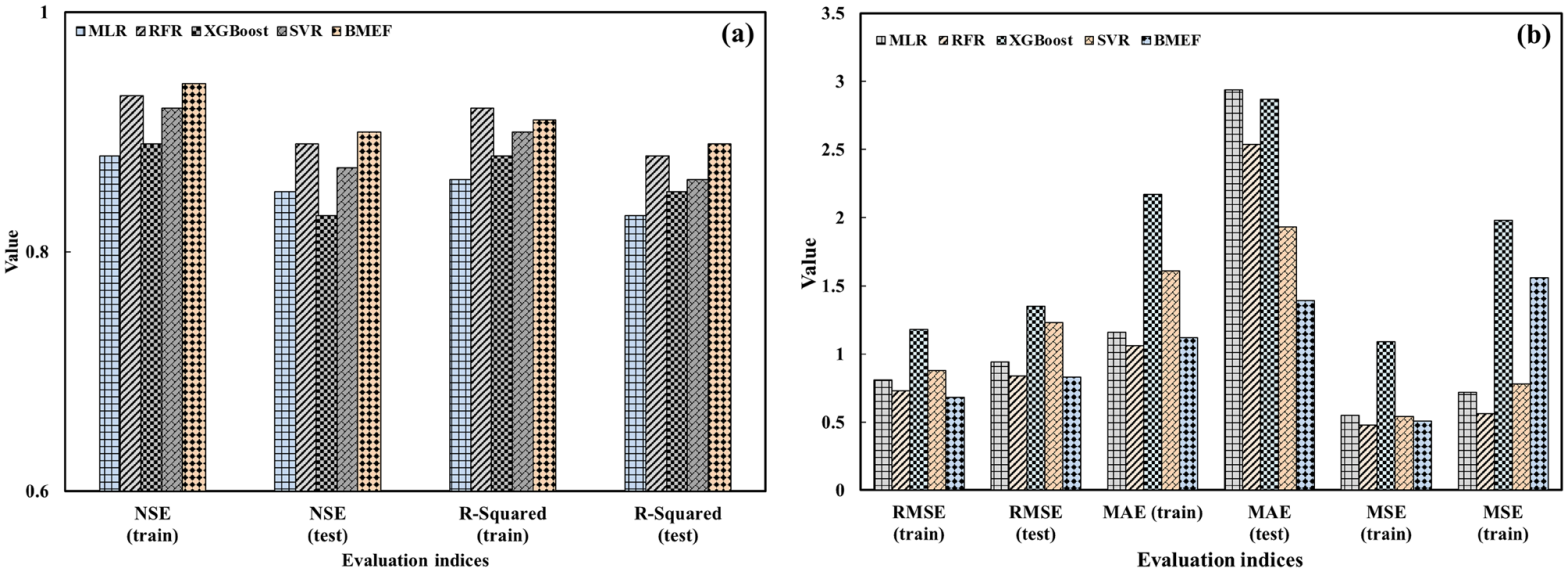
Assessing the effectiveness of MLs and BMEF model for estimation of Chl-a based on (**a**) R-Squared and NSE and (**b**) RMSE, MAE, and MSE for both the training as well as testing period.

Figure 11a and b compare the evaluated indices in the training and testing phase for the ML algorithms. The effectiveness of all the developed algorithms was acquired for each individual ML model and the BMEF model, as shown in Fig. 11 and Table 5. Since S2 does not have a thermal band in scenario 1, considering all bands of S2 as input variables and $R^{2}$ as evaluation criteria, the results obtained for the temperature in the validation/testing period are not satisfied in comparison with other WQPs. Table 6 shows the range of evaluation indices. It may be true that the obtained results indicated that evaluation indices for estimating temperature can classified as an acceptable ML model, but S2 is not reliable to estimate the mentioned parameter compared to other WQIs. Therefore, the results obtained for the estimation of temperature were the least accurate among other WQIs. On the other hand, Chl-a and DO can be estimated using B2, B3, and B4 directly; thus, the obtained results for such parameters are satisfying (Table 6). Additionally, for the validation period, the values of NSE ranged from 0.69 to 0.84 for Chl-a, which had an acceptable rate, thus classifying it as appropriate for the estimating parameters. During both phases, all models have good and acceptable performance due to the ranges of such index between 0.44 and 0.85, for XGBoost to estimate temperature and for BMEF to estimate DO, respectively. R-squared values were classified as satisfactory, good, and very good performance.

**Table 6:** Classification of suitable amount evaluation indices.

Performance rating	NSE	R-Squared	MAE	RMSE
Very good	NSE $\ge$ 0.7	${R}^{2}\ge$ 0.7	MAE < 0.3	RMSE < 0.3
Good	0.5 $\le$ NSE < 0.7	0.5 $\le {R}^{2}$<0.7	0.3 $\le$ MAE < 0.5	0.3 $\le$ RMSE < 0.5
Satisfactory	0.3 $\le$ NSE < 0.5	0.3 $\le {R}^{2}$<0.5	0.5 $\le$ MAE < 0.7	0.5 $\le$ RMSE < 0.7
Unsatisfactory	NSE < 0.3	${R}^{2}$<0.3	MAE $\ge$ 0.7	RMSE $\ge$ 0.7

Taking into account that a lower RMSE value nearer to zero indicates the better effectiveness of the model, the outcomes from both individual models and the BMEF model show that these evaluation indices do not yield an acceptable rate when all bands of S2 data are employed as input variables. For instance, BMEF, the best model, for estimating Chl-a, has an index value of 1.07, while the worst model, XGBoost, for estimating temperature, has a considerably higher index value of 4.01.

**Figure 12:**
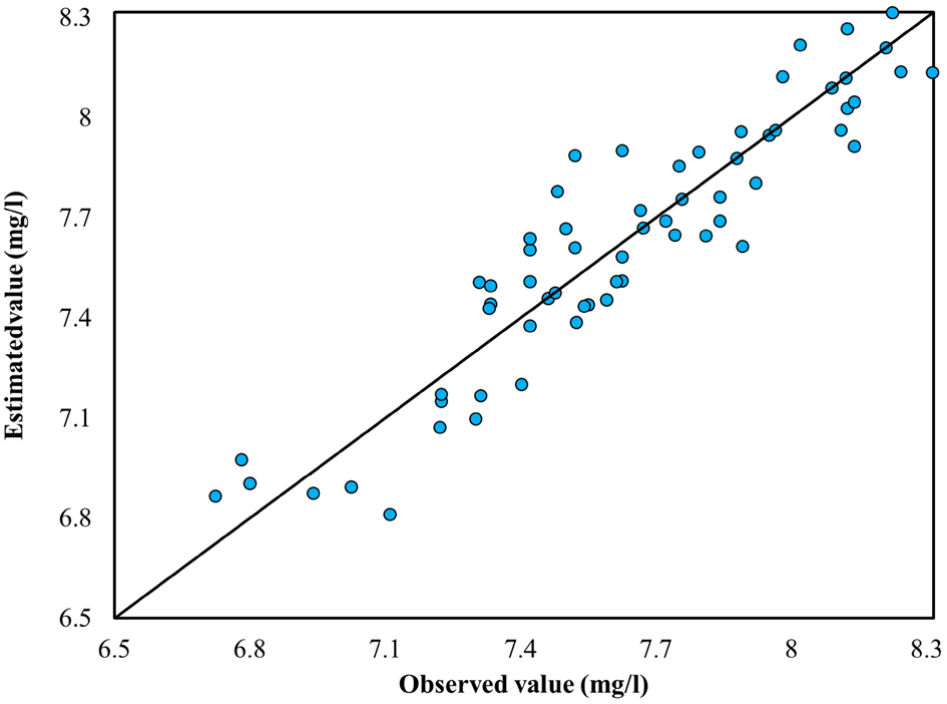
R-Squared of the BMEF model to estimate DO.

Regarding the recorded versus estimated value of WQIs, Fig. 12 illustrates instances where the model exhibited positive errors, indicating underestimation, and, conversely, where negative errors were present, implying an overestimation of the observed values for the WQIs. Additionally, as depicted in Fig. 12, points situated over the 1:1 line indicate that the estimated values held lesser value compared to the recorded values. Furthermore, the closer the scatter values are to the 1:1 line, the better the estimation effectiveness; conversely, the further away the points are from this line, the worse the model's performance.

##### Considering the optimal amalgamation of spectral bands to estimate WQIs

The bands of S2 with a strong correlation with parameters related to WQIs (more than 0.7) are considered as input variables to achieve high accuracy in the predictions. S2 satellites operate in visible, near-infrared, and shortwave infrared bands, mainly used for vegetation mapping, land cover classification, and other applications related to Earth observation. However, these satellites do not have thermal infrared sensors, so they cannot be directly used in temperature measurements. To estimate temperature from S2 data, one would need to use a process known as thermal RS, which involves analyzing thermal infrared data from other sensors or satellite platforms. Therefore, using RGB bands, which have a robust correlation with WQIs (more than 0.7) as input variables to estimate the temperature, leads to satisfactory results. In addition, the results of the best combination of S2 satellite data are presented in Table 7 and Fig. 12.

**Table 7:**
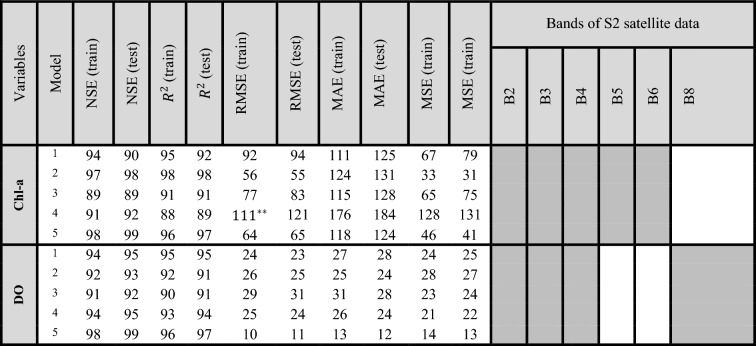
The effectiveness of ML models and the BMEF model regarding all bands of S2 as input variables.

Model 1: Multi-layer Regression. Model 2: RFR. Model 3: XGBoost. Model 4: SVR. Model 5: BME. Temp, temperature. B, band.

*: 0. If the results were less than 100, 0 should be implemented. For example (83 → 0.83).

**: 1. If the results were more than 100, 1 should be implemented. For example (111 → 1.11).

Table 7 illustrates the assessment of analyzed metrics in the training and testing periods for the developed models to estimate the concentration of Chl-a. The BMEF model demonstrates better performance when the Nash–Sutcliffe Coefficient (NSE) and R-squared values are closer to 1. In addition, Table 7 indicates the models’ performance considering RMSE, MAE, and MSE as evaluation indices. Such values had better performance if the value of evaluation indices is closer to zero. Furthermore, a model’s performance can be considered good if NSE is above 0.5 and R-squared is more than 0.5. Hence, in nearly all situations, the BMEF had better performance in comparison with the other developed ML algorithms due to the higher $R^{2}$ and NSE for both calibration and validation periods. It is worth mentioning that since the BMEF model utilizes the best results of individual models, this model performs better in estimating WQIs are better than those of individual ML models. As the RMSE, MSE, and MAE values approach proximity to zero relate to better model had better performance, the outcomes derived from individual models and the BMEF model indicate that when the best combination of S2’s bands are considered as input variables, the level of uncertainty decreases. As a result, the mentioned evaluation indices get closer to 0. As for R-Square, Table 7 shows that the XGBoost model overestimated the observed value due to the high number of points in the testing period above the 45-degree line.

#### General comparison of two scenarios (considering all bands as input variables and the best combination of them)

**Figure 13:**
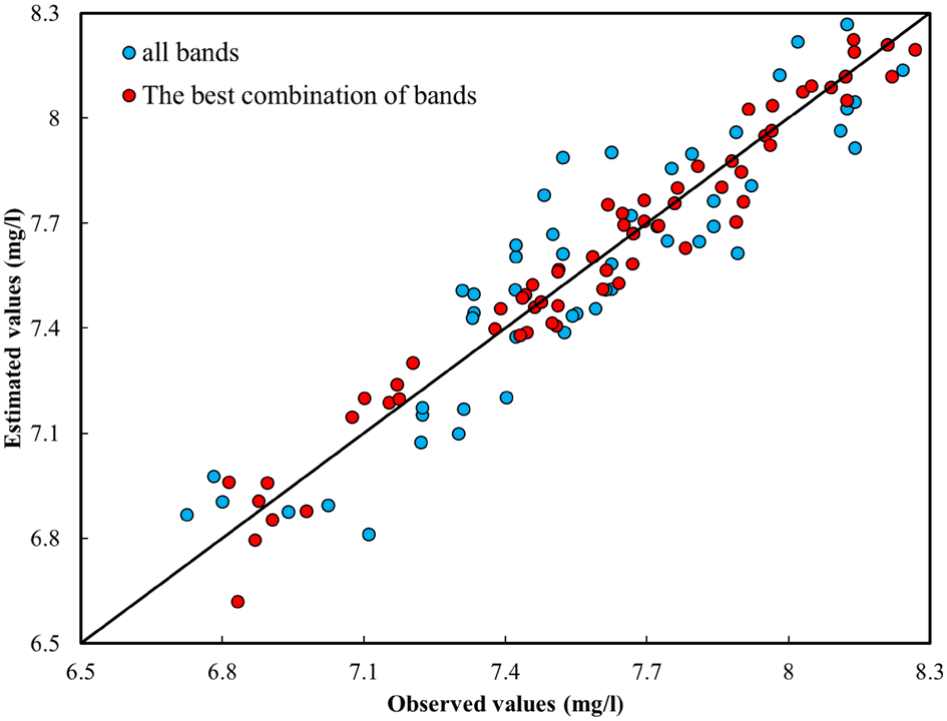
R-Squared data obtained for the BMEF model considering (**a**) all bands as input variables and (**b**) the best combination of bands as input variables for DO concentration.

This section presents the outcomes of all tested algorithms considering the defined scenarios—all bands and the best combination of bands. Figure 13 displays the best results obtained from the BMEF model to estimate WQIs. As such, the blue circles indicate the scenario that considers all bands of S2 as input variables, and the red ones are the scenarios showing the best combination of bands as input variables of the developed models. The findings reveal that the red scatter points, in approximately all WQIs, correlated highly with observed data. For instance, using the best combination of bands (i.e., B2, B3, and B4), as input variables to estimate the amount of Chl-a led to a significant improvement in R-squared, outperforming the results with all bands by 24%, 10%, 27%, 5%, and 10% compared to when all bands are considered as input variables. In all of the comparison scenarios, BMEF was considered as it exhibited the best performance over the individual ML models. In addition, Fig. 15 illustrates the generated spatial water quality maps of WDD using the BMEF model and considering the two defined scenarios to estimate Chl-a. As can be seen from this figure, the similarity between observed data and the scenario when the best combination of bands was considered as input variables is higher than in the other scenario.

Estimating the amount of EC in reservoirs using RS data is a complex task requiring the use of additional data sources and modeling techniques. S2 data cannot directly estimate the amount of EC of water bodies. Therefore, some RS methods have been developed to estimate this parameter, such as a technique that approximates the ratio of bands 4 and 5 (red and red-edge) of S2 data to estimate WQIs.

**Figure 14:**
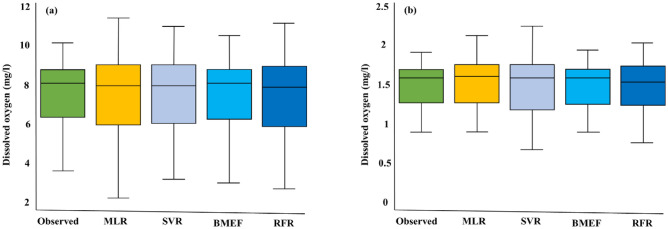
Boxplot for (**a**) dissolved oxygen and (**b**) chlorophyll-a.

Boxplots serve as powerful graphical representations of statistical data, offering insights into the distribution, central tendency, variability, outliers, and skewness within a dataset, thereby facilitating nuanced comparisons and analyses in scientific research. Positioned along the x-axis, the box in a boxplot denotes the median, signified by the line within the box, representing the middle value of the dataset when sorted in ascending order and dividing the data into two halves, each comprising 50% of the points. The length of the box visually illustrates the spread of the central portion of the dataset, delineated by the first quartile (Q1) and third quartile (Q3). Figure 14 illustrates the boxplot of two WQIs, dissolved oxygen (DO) and chlorophyll-a (Chl-a), during the testing phase, offering valuable insights into the distribution of both estimated and recorded values. For instance, in Fig. 14a, the lower band for observed data hovers near 6, contrasting with the range of 6.2–8.3 for the developed models, indicating that the models generally capture the lower range of observed DO concentrations. The upper whisker reveals that while the observed data peaks at 8.3, the models span from 6 to 8.4, encompassing a broad spectrum of values, some of which closely approach the maximum observed values. The median, a representation of the central trend, stands at 7.2 for the observed data and varies from 7.8 to 8.4 for the models, suggesting good alignment between the models’ core values and the observed data's median.

**Figure 15:**
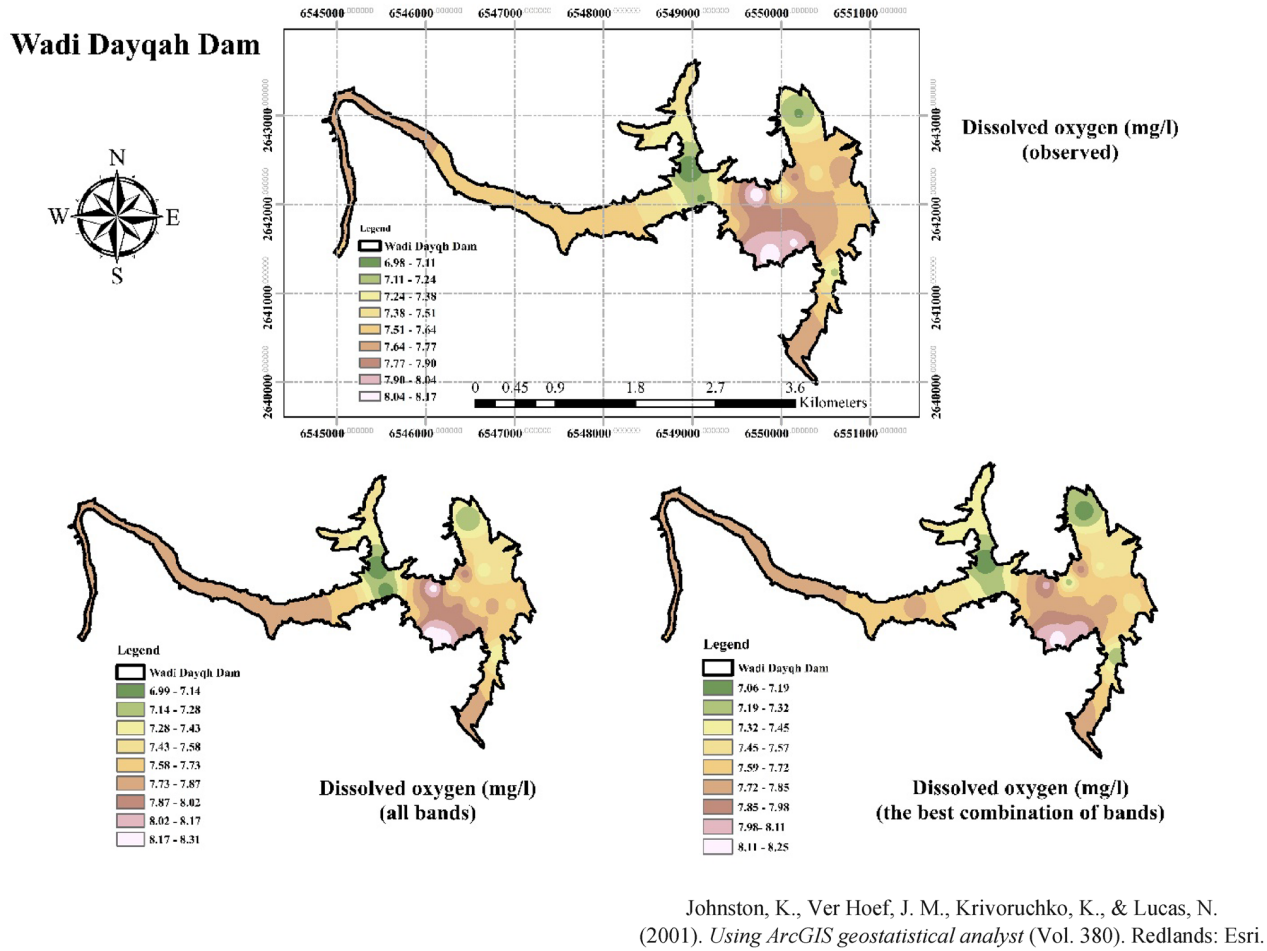
Generated spatial water quality maps of the Wadi Dayqah Dam using the BMEF model (**a**) considering (**a**) all bands as input variables, and (**b**) the best combination of bands as input variables, developed by ArcMap^76^.

Estimating the amount of ORP in reservoirs can be challenging and typically requires more than just spectral band information. Nevertheless, certain spectral bands in S2 data can offer valuable insights related to WQIs, such as the presence of certain algae or suspended particles, which can affect the ORP in reservoirs. The spectral bands that can be useful for this type of analysis are the blue (B2), green (B3), and near-infrared (B8) bands. These bands can be used to calculate various metrics, like the NDVI, NDWI, and TSM index. These indices can offer useful information on the presence of algae, suspended particles, and WQIs in the reservoir. Figure 15 demonstrates the generated spatial water quality maps of DO in WDD.

Estimating the amount of DO in reservoirs using S2 data can be challenging since DO is not directly measurable using optical RS. However, some WQIs that can be divided from S2 data, namely, turbidity, Chl-a, and TDS, can indicate the concentration of DO within the aquatic environment. The bands that can be useful for estimating WQIs related to DO are the blue (B2), green (B3), red (B4), and near-infrared (B8) bands.

### Uncertainty analysis

**Figure 16:**
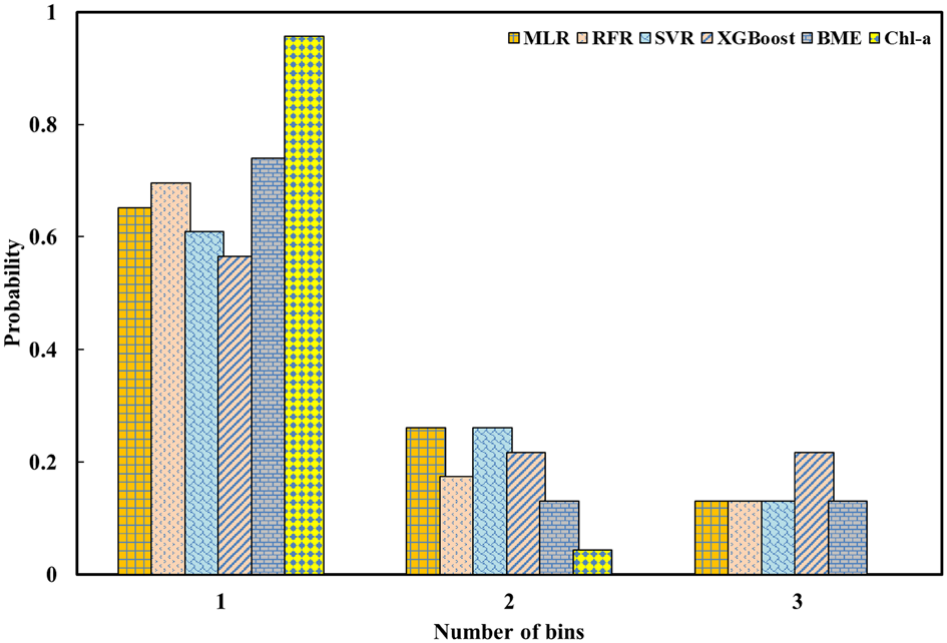
PMF of Chl-a considering all bands as input variables.

This research presents the outcomes of uncertainty analysis using the BMEF modelfor two WQIs—Chl-a and DO. These findings are then juxtaposed with results from the individual ML algorithms. The outcomes of WQI estimation underwent an uncertainty analysis employing a technique rooted in bootstrap resampling and MCA. With this objective in mind, 100 equidistant samples were selected from the original data used for calibration. Subsequently, each set of samples was employed for the calibration of distinct algorithms. Next, the WQIs were determined during the verification phase through the utilization of the calibrated algorithms. Ultimately, the evaluation metric results were harnessed to construct a PMF corresponding to each estimate WQIs. To conduct a more comprehensive investigation, the PMF of ML algorithms to estimate DO and Chl-a are presented hereunder in such a way that two defined scenarios (i.e., using all bands and versus the best combination of bands as input variables). Subsequently, a comprehensive analysis of Chl-a concentration within two scenarios is presented using the optimal model estimator, BMEF. Figure 16 displays the PMF of Chl-a concentration for ML algorithms. It can be seen that BMEF aims to gauge the concentration of Chl-a concentration in the range from zero to one, showing a significant resemblance to the estimation of such WQIs compared to recorded values.

Moreover, both RFR and MLR demonstrate commendable efficacy in approximating the concentration of Chl-a. Conversely, XGBoost does not accurately estimate the Chl-a concentration across various conditions. In summary, BMEF, along with RFR, MLR, SVR, and XGBoost, emerges as the top-performing approaches for Chl-a estimation.

Figure 16 indicates the PMF of the ML algorithms and the BMEF model, considering all bands as predictors for approximating the Chl-a concentration within the WDD. It is worth mentioning that, in this figure, a “bin” refers to a range or interval of values for a continuous variable that is discretized into a finite number of intervals. According to Fig. 16, the BMEF model performed better in comparison with other ML algorithms in estimating the amount of Chl-a in various locations due to the higher height in bins in which observed values were spread over the range. While alternative ML algorithms appear to exhibit a significant degree of concordance with the recorded values, the distribution of observed data across the bins, a characteristic absent in the BMEF model, contributes to this destination. It is also pertinent to note that the same analysis can be concluded when the best band combination is considered input variables.

## Discussions

The findings from this research indicate that spectral-reflectance models coupled with data-fusion techniques can reliably estimate WQIs. The fused spectral data generates models that are capable of estimating WQIs for DO and Chl-a across the entire study region. This method demonstrated significant robustness, producing precise estimation models for various WQIs with algae, sediment, water clarity, and dissolved substances. Abdelmalik utilized advanced spaceborne Thermal Emission and Reflection Radiometer (ASTER) imagery to estimate WQIs in the northeastern region of Egypt^79^, while Mollaee showcased the monitoring of phytoplankton Chl-a concentration in the Western Basin of Lake Erie using RS data. Landsat 8 (L8) has yielded promising outcomes in water quality assessment across various studies^80^, as demonstrated in Mexico^81^ and China^82^. Likewise, S2 data has delivered precise estimations of WQIs, as illustrated in studies conducted in Brazil^83^ and China^84^. Although RS data is extensively utilized in water quality research, the models linking satellite reflectance to in-situ WQI exhibit significant variation. Hence, the outcomes have varied in accuracy depending on the location and the specific modelling approach employed^85^. This challenge arises in part from the inconsistency among studies in determining which spectral features or bands are valuable for establishing functional relationships between WQIs and reflectance data obtained from satellite imagery^86,87^. However, ML models like RF, support vector machine (SVM), ANN, etc., have demonstrated superior accuracies thanks to their capability to autonomously learn from data, uncover concealed patterns, and handle the non-linearity inherent in reflectance data and optically-active WQIs. Therefore, the selection of satellite image data and modelling techniques is crucial for enhancing the adaptive capacity of water quality monitoring for the WDD. Moreover, relying solely on mode-based pixel-level estimation of WQIs to assess water quality status is highly uncertain, as errors within the models can propagate to the population level. Nevertheless, integrating probability samples from the sampling design with robust modeling can yield reliable point estimates and facilitate spatio-temporal mapping of WQIs within the framework of model-assisted estimation^88^.

## Conclusions

The current study proposed a novel technique to enhance water quality estimation outcomes by developing a fusion model based on the BMEF model that combines ML as well as RS. The motivation for developing such a concept was to achieve more accuracy and less uncertainty related to predictions made by specific models. The effectiveness of the suggested technique was assessed by estimating WQIs in the northeast of Oman. As the study primarily aimed to develop a model to estimate the number of various WQIs, including Chl-a, ORP, DO, EC, and pH, the input variables of the ML and BMEF models were derived from S2 bands. Therefore, four ML models, namely MLR, RFR, SVR, and XGBoost model, were employed to estimate WQIs. The primary purpose of BMEF is to enhance the results obtained from singular ML models; therefore, the best results obtained from each model were combined together. Then, two scenarios were evaluated to estimate WQIs: 1. employing all bands of S2 and 2. Using the best combination of S2 bands. Results obtained from the developed ML and BMEF models indicated that when the best combination of various bands was considered as input variables, better results were obtained compared to the use of all S2 bands.

According to the outcomes of the uncertainty analysis, the BMEF procedure produced more accurate results than individual models. Moreover, it was concluded that the same or even better results can be obtained by combining the bands that highly influence the results for each WQI. As a result, improving estimation accuracy does not always originate from the inclusion of additional variables in the inputs. The BMEF model, which employs RS data, can be used to estimate how WQIs can benefit various groups of people, including water utilities, environmental agencies, policymakers, and the general public. For example, water utilities can make use of these models to improve their treatment processes, decrease expenses, and comply with regulations. In forthcoming works, the enhanced projections produced by the BMEF model can enhance the development of improved water quality management.

## Data Availability

The water quality data of this study is related to the Wadi Dayqah Dam, located in Oman, which was obtained using a CTD device and is not publicly available. However, upon reasonable request subject to any applicable restrictions, the corresponding authors can provide the data used in this study.

## Abbreviations

AI: Artificial intelligence
ANNs: Artificial neural networks
BMEF: Bayesian maximum entropy-based fusion
Chl-a: Chlorophyll-a
C-V: Cross-validation
CTD: Conductivity, temperature, depth
DO: Dissolved oxygen
EC: Electrical conductivity
MAE: Mean absolute error
MC: Monte Carlo
MCA: Monte Carlo analysis
MF: Mathematical function
ML: Machine learning
MLR: Multilayer regression
MSE: Mean square error
NDVI: Normalized difference vegetation index
NDWI: Normalized difference water index
NSC: Nash Sutcliffe
ORP: Oxidation–reduction potential
PMF: Probability mass function
RF: Random forest
RFR: Random forest regression
RMSE: Root mean squared error
RS: Remote sensing
R-Squared: Coefficient of determination
SIAC: Sensor independent atmospheric correction
SVR: Support vector regression
S2: Sentinel-2
TSM: Total suspended matter
WDD: Wadi Dayqah dam
WQIs: Water quality indicators
